# A comparative analysis of EGFR-targeting antibodies for gold nanoparticle CT imaging of lung cancer

**DOI:** 10.1371/journal.pone.0206950

**Published:** 2018-11-08

**Authors:** Jeffrey R. Ashton, Elizabeth B. Gottlin, Edward F. Patz, Jennifer L. West, Cristian T. Badea

**Affiliations:** 1 Department of Biomedical Engineering, Duke University, Durham, North Carolina, United States of America; 2 Department of Radiology, Duke University Medical Center, Durham, North Carolina, United States of America; 3 Department of Pharmacology and Cancer Biology, Duke University Medical Center, Durham, North Carolina, United States of America; Case Western Reserve University, UNITED STATES

## Abstract

Computed tomography (CT) is the standard imaging test used for the screening and assessment of suspected lung cancer, but distinguishing malignant from benign nodules by CT is an ongoing challenge. Consequently, a large number of avoidable invasive procedures are performed on patients with benign nodules in order to exclude malignancy. Improving cancer discrimination by non-invasive imaging could reduce the need for invasive diagnostics. In this work we focus on developing a gold nanoparticle contrast agent that targets the epidermal growth factor receptor (EGFR), which is expressed on the cell surface of most lung adenocarcinomas. Three different contrast agents were compared for their tumor targeting effectiveness: non-targeted nanoparticles, nanoparticles conjugated with full-sized anti-EGFR antibodies (cetuximab), and nanoparticles conjugated with a single-domain llama-derived anti-EGFR antibody, which is smaller than the cetuximab, but has a lower binding affinity. Nanoparticle targeting effectiveness was evaluated in vitro by EGFR-binding assays and in cell culture with A431 cells, which highly express EGFR. In vivo CT imaging performance was evaluated in both C57BL/6 mice and in nude mice with A431 subcutaneous tumors. The cetuximab nanoparticles had a significantly shorter blood residence time than either the non-targeted or the single-domain antibody nanoparticles. All of the nanoparticle contrast agents demonstrated tumor accumulation; however, the cetuximab-targeted group had significantly higher tumor gold accumulation than the other two groups, which were statistically indistinguishable from one another. In this study we found that the relative binding affinity of the targeting ligands had more of an effect on tumor accumulation than the circulation half life of the nanoparticles. This study provides useful insight into targeted nanoparticle design and demonstrates that nanoparticle contrast agents can be used to detect tumor receptor overexpression. Combining receptor status data with traditional imaging characteristics has the potential for better differentiation of malignant lung tumors from benign lesions.

## Introduction

Lung cancer is the leading cause of cancer death worldwide and the number of deaths attributed to lung cancer is expected to continue increasing[1]. Early detection of lung cancer is critical for reducing mortality rates. Computed tomography (CT) imaging is well-suited for the detection of lung cancer due to the high inherent contrast between the normal air-filled lungs and soft tissue masses. In the National Lung Cancer Screening Trial (NLCST), CT screening in high-risk patients reduced lung cancer-specific mortality[2], and the United States Preventative Services Task Force now recommends routine CT lung cancer screening for high risk patients (age 55–80 with a significant history of smoking). Although CT lung cancer screening is very sensitive for detecting pulmonary nodules and masses, the differentiation of malignant nodules from benign nodules based on CT morphology alone is challenging[3]. Consequently, a large number of invasive procedures are performed on patients with benign nodules in order to exclude malignancy. In the NLCST, 25% of the trial’s surgical procedures were performed on nodules that turned out to be benign. Although positron emission tomography (PET) imaging can be used to stratify the risk of suspicious nodules, the spatial resolution of PET is not sufficient to adequately characterize sub-centimeter nodules[4]. There is a clear need to improve CT imaging for non-invasive lung nodule characterization. This aim can be achieved by expanding the role of CT beyond its present anatomical imaging capabilities towards functional and molecular-based imaging. One potential method for improving discrimination between benign nodules and malignant tumors is to target cell-surface receptors that are present on malignant cells, but not benign cells. Contrast agents that are targeted to those specific cell-surface receptors should demonstrate increased enhancement in regions with high receptor expression. The development of targeted contrast agents has the potential to improve lung cancer detection and characterization.

In this work, we focus on developing a nanoparticle contrast agent that targets the epidermal growth factor receptor (EGFR). EGFR belongs to a family of receptor tyrosine kinases that trigger an array of signaling pathways that lead to cell growth, proliferation and survival. Upregulation or oncogenic activation of EGFR can lead to uncontrolled growth and tumor progression. 10–15% of Caucasians and up to 50% of Asians with lung adenocarcinoma have an activating mutation in the EGFR gene[5], and approximately 93% of adenocarcinomas demonstrate EGFR expression[6], with 40–80% of adenocarcinomas exhibiting overexpression of EGFR[5, 7]. Most tissues (including benign tumor nodules) have a low basal level of EGFR expression, so targeting EGFR-expressing lung tumors can be an effective strategy. Because of the important role that EGFR plays in many lung adenocarcinomas, several drugs have been developed that specifically target the EGFR pathway, including EGFR-inhibiting antibodies (cetuximab) and small molecule tyrosine kinase inhibitors (erlotinib, gefitinib, afatinib). These drugs are not effective for all patients overexpressing EGFR, but are highly effective in most patients that harbor an activating mutation in the EGFR gene[5].

In this study, gold nanoparticles were used as the EGFR-targeted CT contrast agent. The experimental use of nanoparticles as CT contrast agents has been increasing in popularity over the last decade[8]. Gold nanoparticles, in particular, are well-suited for use as CT contrast agents because they have high x-ray attenuation compared to iodine (~2.7 times higher per mass), they exhibit high in vivo stability, and their surface can be readily modified to render them biocompatible and target them to a variety of in vivo biomarkers[9–13]. Gold nanoparticles have been used as a micro-CT contrast agent for the targeting of multiple tumor markers in mice, including Her2[14], the gastrin-releasing peptide (GRP) receptor[15], the folic acid receptor (FAR)[16], the LDL receptor [17], and tumor microcalcifications[18]. A previous small-scale study has also demonstrated initial feasibility of targeting EGFR with gold nanoparticles[19].

Several obstacles need to be considered for the successful use of targeted nanoparticle probes as contrast agents. Nanoparticles naturally accumulate in tumors due to the enhanced permeability and retention effect (EPR)[20], which makes them excellent probes for tumor imaging. However, because nanoparticle accumulation in tumors due to EPR is so effective it is difficult to demonstrate additional improvement in accumulation with targeted probes. Studies have shown that there is no benefit to adding targeting molecules to large-sized (>100 nm) nanoparticles, while smaller particles show increased retention in tumor tissues when they are actively targeted to a receptor[21]. Presumably, this is because large particles are naturally retained in the tumor tissue at higher levels than smaller, more mobile particles. Therefore, active targeting of smaller particles allows them to have tumor retention similar to that of large nanoparticles, but with less non-specific retention. This problem requires optimization to balance the efficacy of EPR (increased for larger size) with the specificity benefit of active targeting (increased for smaller particles). Delivery of nanoparticles to the center of tumors is also a difficult problem for larger nanoparticles. While the periphery of tumors is usually very well perfused, and the majority of nanoparticles accumulate in the periphery, the center tends to be poorly perfused. Large nanoparticles and macromolecules do not diffuse through the tissue well enough to reach the center of the tumor. For these reasons, we use relatively small nanoparticles (25 nm) in this study to try to maximize the sensitivity and specificity for lung tumor targeting.

Another major challenge for targeted probes is immune recognition. Stealth nanoparticles which are coated with a surface layer of poly(ethylene glycol) (PEG) tend to resist protein adsorption and are therefore not immediately recognized by phagocytic cells[22]. However, immobilized proteins (especially antibodies), situated on the surface of the nanoparticle to provide tumor specificity, are sites of immune recognition. The Fc portion of antibodies is particularly problematic, as phagocytic cells have cellular receptors that bind to the Fc domain, leading to phagocytosis[23]. The Fc domain can also activate the complement cascade, leading to deposition of opsonins, immune recognition and immune activation. This recognition can lead to rapid clearance of antibody-coated nanoparticles from the bloodstream, which reduces tumor uptake[24]. Alternatives to full antibody targeting of tumors include the use of aptamers, small molecules (e.g. folic acid), or antibody fragments that lack the Fc domain.

In this study we compare the results of targeting using a full-sized anti-EGFR antibody (cetuximab, C225) with targeting using a llama-derived single domain antibody. Llama heavy chain variable region antibody fragments (VHH domains) are the smallest identified antibody fragments that retain target specificity because they are only composed of a single binding domain. EGFR-specific VHH domains have been produced and characterized previously[25]. Because of their smaller size, VHH domains infiltrate tumors more rapidly than monoclonal antibodies, are highly specific, and are reported to be non-immunogenic in both mice and humans[26]. These domains have high affinity for their targets, although usually somewhat lower affinity than many multi-domain human antibodies. We hypothesized that including these VHH domains on the nanoparticle surface would decrease nanoparticle immune recognition compared to full-sized antibodies due to their lack of the Fc fragment, which would prolong the blood residence time of VHH-targeted nanoparticles. The smaller antibody fragments can also pack much more densely on the surface of the particle than a full-sized antibody, which could potentially create a particle with high avidity for EGFR. The effectiveness of VHH-targeted gold nanoparticles was compared to targeting with a clinically-used monoclonal whole antibody, C225 (cetuximab). This antibody is approved for the treatment of EGFR-positive tumors and has very high binding affinity (~0.39 nM) for EGFR[27]. The VHH-122 antibodies have lower binding affinity (~40 nM)[25], but we expected that the higher avidity and reduced clearance might be able to make up for this lower binding affinity. In this study, the targeting effectiveness of gold nanoparticles conjugated with both antibody types (as well as non-targeted controls) was tested in vitro as well as in vivo in an EGFR-overexpressing mouse tumor model. Dual-energy CT was used to detect and measure concentrations of gold nanoparticles within tumor-bearing mice. We have previously shown the ability to accurately measure concentrations of gold and iodine using dual-energy CT both in vitro and in vivo[28, 29]. This accurate in vivo measurement of nanoparticle uptake allowed for direct comparisons between the tumor uptake of the targeted and non-targeted gold nanoparticles. The results of this study can be used to better guide the development of targeted agents that one day can be used to improve the detection and accurate diagnosis of lung cancer.

## Materials and methods

### Gold nanoparticle synthesis

25 nm gold nanoparticles were synthesized via the citrate reduction of gold chloride salt using a modified Frens methodP[30]. 50.55 mL of HAuClR_4_R (10 mg/mL) was added to 1.5 L of ultrapure water and the solution was brought to boiling. 9 mL of a pre-warmed sodium citrate solution (100 mg/mL) was rapidly injected into the gold solution under vigorous mixing, after which the solution quickly changed color from yellow to deep red. The solution was kept at 100°C and mixed for an additional 15 minutes to complete the reaction, and then the solution was cooled to room temperature. The nanoparticles were then passed through a 0.22 μm filter to remove any aggregates that may have formed during the reaction. The size and distribution of the nanoparticles were determined by transmission electron microscopy (TEM) using an FEI Tecnai G^2^ Twin TEM (FEI, OR) operating at 200 mV.

### Antibody preparation

An EGFR-specific VHH domain (VHH-122) was chosen from a llama-VHH domain library, as described previously[25]. VHH-122 was expressed in E.coli HB2151 periplasm and then purified by nickel affinity chromatography using His SpinTrap columns (GE Healthcare) according to the manufacturer’s instructions. This specific VHH domain has been well-characterized[25], and has been shown to bind to both mutated and wild-type EGFR extra-cellular domain with roughly equal affinity, approximately 40 nM. VHH-122 has a molecular weight of 15.4 kDa.

A full-sized chimeric (human/mouse) clinically-used monoclonal antibody against EGFR (C225, cetuximab) was purchased from the manufacturer (Lilly). C225 has a binding affinity of 0.39 nM for the EGFR extracellular domain and has an approximate molecular weight of 152 kDa[31].

### Targeted nanoparticle preparation

VHH-122 and C225 antibodies were linked to gold nanoparticles via a PEG linker molecule. The PEG molecule contained a reactive NHS ester group (succinimidyl valerate, SVA) capable of forming covalent bonds with primary amines on the antibodies. The other end of the PEG molecule contained a thiol group (orthopyridyl disulfide, OPSS) which can bind to the gold nanoparticle surface. The antibodies were PEGylated by reacting the OPSS-PEG-SVA (5 kDa, Laysan Bio) with the antibodies at a ratio of 20:1 (PEG:antibody) for VHH-122 and 2.5:1 for C225. The reaction was performed in HEPES-buffered saline at pH 8.6 at 4°C overnight. The PEG-antibodies were then mixed with bare gold nanoparticles at a ratio of 50 antibodies/particle for VHH-122 or 10 antibodies/particle for C225. These ratios were chosen based on preliminary testing that showed particle aggregation during the surface conjugation when higher concentrations of protein were added to the surface. These nanoparticles were rocked at 4°C for 2 hours, after which methoxy PEG thiol (mPEG-SH, 5 kDa) was added to the gold nanoparticles in large excess to fill in any gaps remaining on the surface after antibody conjugation. Control (non-targeted) nanoparticles were also produced by adding a large excess of mPEG-SH (5 kDa) to bare gold nanoparticles in the absence of any antibodies. All gold nanoparticles were rocked overnight at 4°C after PEG addition and then rinsed by centrifugation. The nanoparticles were filter-sterilized and concentrated prior to use.

### Targeted nanoparticle characterization

The size distribution and surface charge of the gold nanoparticles (bare, PEGylated, and antibody-conjugated) were characterized by dynamic light scattering (DLS) and zeta potential using a Zetasizer Nanoseries (Malvern, UK) at 25°C. Nanoparticles were tested for stability in physiological solutions by incubating them in phosphate-buffered saline (PBS) and culture medium (Dulbecco’s Modified Eagle’s Medium, DMEM) with 10% fetal bovine serum (FBS). Resistance to aggregation was determined by measuring the peak optical absorbance at the nanoparticle surface plasmon resonance peak wavelength using a Cary 50 UV-Vis spectrophotometer (Varian, CA), while size change due to the adsorption of serum proteins was tracked by serial DLS size measurements.

Conjugation of the antibodies to the AuNPs was further confirmed and quantified by gel electrophoresis (SDS-PAGE) analysis. After rinsing the targeted AuNPs to remove unbound proteins, the bound antibodies were released from the gold surface by reaction with dithiothreitol (DTT)[32]. SDS was then added to the solution and the solution was heated to 95°C for 15 minutes to fully denature the proteins. The nanoparticles were then pelleted by centrifugation and the supernatant containing the denatured antibodies was loaded onto an SDS-PAGE gel along with VHH-122 and C225 standards and PEGylated antibody standards of known concentration. The gels were stained with Oriole fluorescent dye (Bio-Rad) according to the manufacturer’s instructions. The amount of antibody in each lane was quantified by comparing the band intensity in the unknown lanes to the intensity of the known standards. The amount of antibody on each nanoparticle was then calculated by using the concentration of AuNPs in the original samples. AuNP concentration was determined based on UV-Vis measurements of optical absorbance at 450 nm using a published extinction coefficient for 25 nm spherical AuNPs[33].

To verify that the protein conjugation to the nanoparticle surface was via the gold-thiol bond, rather than non-specific protein adsorption on the surface, some bare nanoparticles were conjugated with non-PEGylated VHH-122 and C225 antibodies, followed by the addition of mPEG-SH as described above. Gel electrophoresis analysis was repeated on these samples to quantify the amount of protein attached to the nanoparticle surface non-specifically.

### In vitro EGFR binding assay

Binding of EGFR to the antibody-conjugated AuNPs was assessed by performing an enzyme-linked immunosorbent assay (ELISA) in the nanoparticle suspension. Antibody-conjugated AuNPs were blocked with 10% donkey serum in PBS, then incubated with a soluble recombinant human EGFR extracellular domain-Fc chimera (Sino Biological) at 10 nM in blocking buffer for 2 hours. The nanoparticles were then rinsed by repeated rounds of centrifugation and resuspension in PBS. Binding of soluble EGFR to the gold nanoparticles was then detected by adding a horseradish peroxidase-conjugated anti-human IgG antibody, followed by another round of rinsing. 3,3′,5,5′-Tetramethylbenzidine (TMB) substrate was added and the reaction was stopped by concentrated HR_2_RSOR_4_R after 15 minutes. The suspensions were then centrifuged to pellet the nanoparticles and the absorbance of each supernatant at 450 nm was measured using a plate reader in order to quantify the amount of EGFR bound to the nanoparticles in the suspension. The ELISA measurements were normalized to both nanoparticle concentration and total antibody concentration in the original sample.

### In vitro cell targeting

In vitro cell targeting studies were performed in culture using A431 cells (from ATCC #CRL-1555). The A431 cell line is derived from a human epidermoid carcinoma and is characterized by very high surface expression of the wild type EGFR, estimated at approximately 5x10^5^ copies of EGFR per cell when grown in culture[34]. A431 cells were cultured in Improved MEM Zinc Option Media (Gibco) supplemented with 10% FBS in 4 well chamber slides to 50% confluence, then AuNPs (2 mg/mL in 10% FBS and culture media) were added to the chambers and incubated overnight. The slides were then rinsed gently two times with culture media and two times with PBS to remove unbound AuNPs. Half of the slides were fixed with 4% paraformaldehyde and used for microscopy, while the other half was used for gold quantification by inductively-coupled plasma optical emission spectroscopy (ICP-OES).

Fixed slides were imaged using a Zeiss Axiovert 135 inverted microscope (Carl Zeiss Inc., Thornwood, NY) and a Cytoviva darkfield condenser (CytoViva, Inc., Auburn, AL). All images were acquired at 200x magnification. Darkfield images were taken of each cell culture sample to detect gold nanoparticles bound to the cells. With darkfield imaging, indirect sample illumination enables image production from light scattered by the samples. Gold nanoparticles are readily detected due to their increased scattering properties relative to the surrounding tissue.

For ICP-OES analysis, cells from each sample group were scraped off the slides and collected in a microcentrifuge tube. The cells were then pelleted by centrifugation and the supernatant was removed. Cell pellets were digested in trace metals grade aqua regia for 72 hours. The samples were then filtered through a 0.45-μm filter and diluted with 2% nitric acid to 10 mL. Standard solutions of known gold concentrations (8 concentrations ranging from 0.1–10 ppm) were prepared in 2% aqua regia using certified reference material Gold Standard for ICP (Fluka Analytical). The standards and digested samples were analyzed for gold concentration by ICP-OES (Teledyne Leeman Laboratories, Hudson, NH).

### Animal research ethics statement

All animals were handled in accordance with good animal practice as defined by the relevant national and/or local animal welfare bodies, and all animal work was approved by the Institutional Animal Care and Use Committee (IACUC protocol A283-11-11) of Duke University Medical Center. The Duke University Medical Center animal management program is accredited by the American Association for the Accreditation of Laboratory Animal Care and meets National Institute of Health standards as set forth in the ‘‘Guide for the Care and Use of Laboratory Animals” (DHHS Publication No. (NIH) 85–23, Revised 1985). The institution also accepts as mandatory the PHS ‘‘Policy on Humane Care and Use of Laboratory Animals by Awardee Institutions” and ‘‘NIH Principles for the Utilization and Care of Vertebrate Animals Used in Testing, Research and Training”. Mice were anesthetized for all procedures and imaging using inhaled 4% isoflurane. Mice were housed in ventilated cages with five single-gender mice per cage. Nude mice additionally had sterilized bedding, food and water. All mice had free access to food and water and regular light/dark cycles. Mouse tumors were monitored by caliper measurement 3 times per week. Mice were euthanized at humane endpoints (tumor size > 1.5 cc, tumor ulceration, or tumor affecting function). At the end of the studies, the mice were euthanized by inhaled isoflurane.

### In vivo imaging–biodistribution study

For the initial biodistribution imaging study, 3 groups of healthy C57BL/6 mice (n = 3 in each group) were injected by tail vein with C225-AuNPs, VHH-AuNPs or PEG-AuNPs (150 mg gold/mL in PBS, 20 mg/25 g body weight). CT imaging was performed at 0, 2, 4, 12, 24, and 36 hours post-injection to track the dynamic gold nanoparticle concentration. CT enhancement was measured in the blood, liver, spleen and kidney at each time point.

### In vivo imaging–tumor targeting study

For the tumor targeting study, 3 groups of 4–6 week old female athymic nude mice (n = 8 in each group) were injected subcutaneously in the flank bilaterally with 1x10P^6^P A431cells in 100 uL Matrigel (Corning). After tumors reached 0.5 cc (10–14 days after injection), mice were injected by tail vein with one of three contrast agents: C225-AuNPs, VHH-AuNPs, or PEG-AuNPs (150 mg gold/mL in PBS, 20 mg/25 g body weight). Dual-energy CT images were acquired immediately after injection and 48 hours post-injection. Gold concentrations were quantified within the tumors at each time point.

### Dual-energy micro-CT system

All CT imaging for this study was performed using a custom-built dual source micro-CT system[35] with two x-ray tubes and two detectors arranged orthogonally around a vertical rotating small animal cradle. The mice were scanned while free breathing under anesthesia using 2–3% isoflurane delivered by nose-cone. A pneumatic pillow positioned on the animals' thorax connected to a pressure transducer was used to monitor breathing, and body temperature was maintained with heat lamps, a rectal probe, and a feedback controller. The scanning parameters for the dual-energy scans were: 80 kVp, 160 mA, 10 ms/exposure for the first imaging chain and 40 kVp, 250 mA, 16 ms/exposure for the second imaging chain. Projections from the two imaging chains were acquired simultaneously at each angle of rotation. A total of 360 views were acquired for each chain over a full 360° rotation.

### CT post-processing and dual-energy gold quantification

CT image post-processing was performed as described previously[28, 29]. Raw 40 kVp and 80 kVp CT datasets were reconstructed using the Feldkamp algorithm[36] with an 88 μm isotropic voxel size. Affine registration was performed between corresponding 40 and 80 kVp reconstructed volumes and then each dataset was denoised using joint bilateral filtration (BF) to improve the material decomposition[37, 38]. Dual-energy decomposition was performed by using a CT sensitivity matrix derived from gold concentration standards, which we have previously validated both in vitro and in vivo[28, 29, 39]. The result of the dual-energy decomposition was a gold concentration map, in which intensity values represent the calculated concentration of gold within each voxel.

### CT image analysis

CT images were analyzed using Avizo (FEI Visualization Sciences Group, Burlington, MA). Regions of interest including the blood, liver, spleen, and kidneys (for the biodistribution study) and the tumors (for the targeting study) were segmented in Avizo. Because of the high gold concentrations in the blood, liver, and spleen, these organs were readily segmented using the software’s automatic segmentation tools. The tumors and kidneys were segmented by manually drawing rough regions around the region of interest, then thresholding the regions until non-enhancing tissues outside the kidney or tumor were completely excluded. The concentration of gold in each region of interest was measured for each timepoint. For tumor gold quantification, the tumor background signal was subtracted from the measured signal in the enhancing regions before measuring the gold concentration. Nanoparticle blood half-life was calculated for each nanoparticle type by fitting the blood gold concentration data from the biodistribution study to an exponential decay curve.

### Statistics

Statistical differences between the PEG, VHH-122 and C225 groups in each study were tested using a one-way ANOVA followed by a post-hoc Tukey test for individual comparisons. Statistical significance was noted at the p<0.05 level for all comparisons (and in all figures). Error bars in all plots represent the standard error of the mean.

## Results

### Nanoparticle characterization

**Fig 1:**
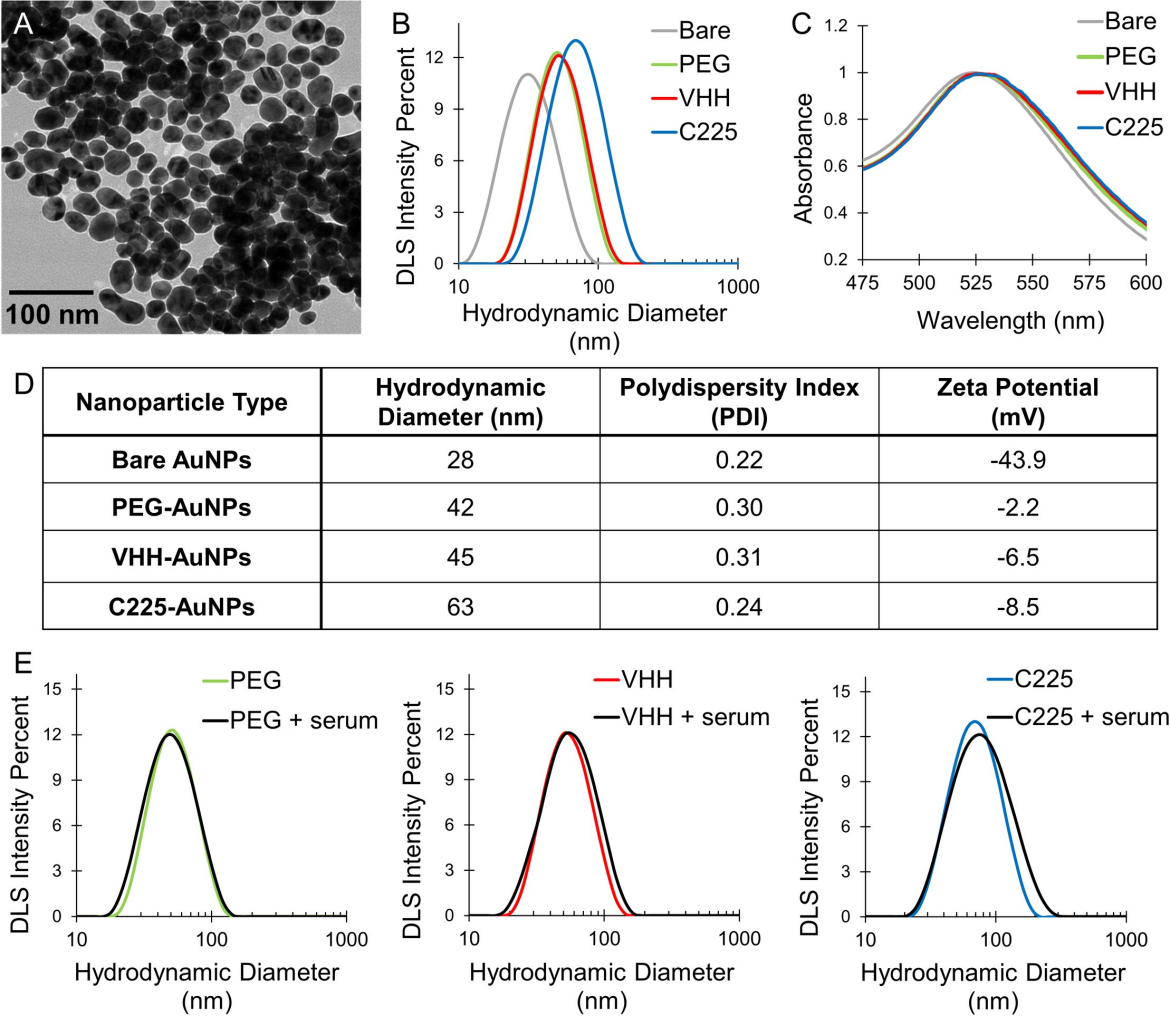
Gold nanoparticle characterization. (A) TEM image of bare gold nanoparticles. (B) DLS plots of nanoparticle hydrodynamic diameter before after surface modification. (C) UV-VIS spectroscopy of gold nanoparticles, showing a shift in peak absorbance as a result of surface modification. (D) DLS measurements of average hydrodynamic diameter, polydispersity index, and zeta potential of nanoparticles with each surface modification. (E) Hydrodynamic diameter measurements of PEG, VHH, and C225-AuNPs before and after incubation with 10% fetal bovine serum for 72 hours.

Gold nanoparticle characterization is shown in Fig 1. TEM images of the bare AuNPs (see Fig 1A) showed spherical gold nanoparticles with an average diameter of 25±4 nm and average aspect ratio of 1.2. The average hydrodynamic diameter (see Fig 1B) increased from 28 nm for the bare nanoparticles to 42 nm after PEGylation. The addition of VHH-122 antibodies increased the average diameter only marginally (45 nm) compared to PEG alone. The addition of C225, on the other hand, increased the hydrodynamic diameter of the nanoparticles to 68 nm because of the larger size of the IgG antibodies. The polydispersity index (see Fig 1D) for all the nanoparticles types was comparable (0.22–0.31). Zeta potential measurements showed an average zeta potential of -41 mV for the bare nanoparticles, which increased to >-10 mV for all the modified nanoparticles, showing effective surface passivation and improved biocompatibility compared to the bare nanoparticles. UV-VIS spectroscopy (see Fig 1C) showed a shift in the peak optical absorbance (surface plasmon resonance peak) from 523 nm for the bare nanoparticles to 525 nm for the PEG-AuNPs and VHH-AuNPs and to 526 nm for the C225-AuNPs. This shift is due to a change in the local refractive index of the gold nanoparticle surface when the chemical composition on the surface is altered. The small shifts seen here show that molecules are binding to the surface, but the change in nanoparticle surface properties is not causing nanoparticle aggregation, which would show a much larger shift in the absorption peak.

Aggregation studies showed that PEG-AuNPs, VHH-AuNPs and C225-AuNPs were stable for > 6 hours (no measurable change in peak absorbance by UV-Vis) in both physiological saline (PBS) and DMEM growth medium + 10% FBS, indicating both stability to particle aggregation and resistance to protein adsorption and resultant particle cross-linking and aggregation in serum. Bare nanoparticles aggregated and sedimented out of suspension within 10 minutes of exposure to PBS. Serial DLS size measurements (see Fig 1E) showed that the PEG-AuNPs and VHH-AuNPs had no change in hydrodynamic diameter after 72 hours of incubation in 10% FBS, demonstrating strong resistance to protein adsorption. The C225-AuNPs hydrodynamic diameter increased slightly after 72 hours in 10% serum, from 63 nm to 69 nm. This shows that these nanoparticles with large antibodies on the surface had a small amount of protein adsorption occur over the course of 3 days in serum. However, this was still a relatively small change and no aggregation was demonstrated as a result of the protein interactions.

SDS-PAGE quantification of antibodies on the nanoparticle surface following conjugation showed that on average there were ~25 VHH-122 antibodies and ~5 C225 antibodies bound per 25 nm nanoparticle (see S1 Dataset). To demonstrate that this linkage was specifically through the PEG-linker (and not through non-specific protein-surface interactions), some bare nanoparticles were conjugated with non-PEGylated antibodies, followed by the addition of mPEG-SH as described. Quantification of antibodies of the surface of these nanoparticles showed that the mass of protein bound to the surface was undetectable for both the VHH-122 and C225 antibodies. This demonstrates that the antibodies are only able to form a stable bond with the gold surface when the PEG-linker is present.

### In vitro nanoparticle EGFR binding

**Fig 2:**
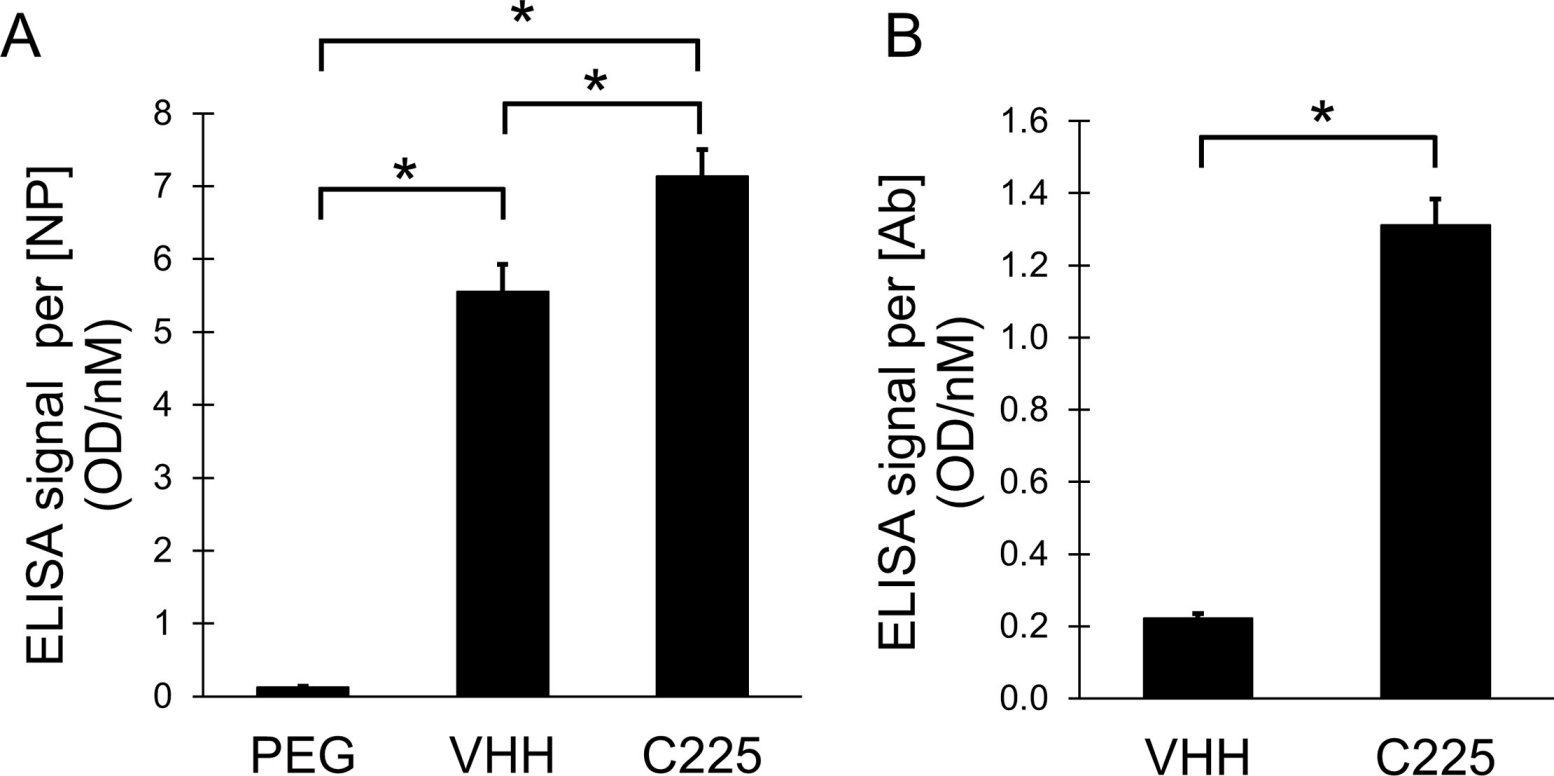
ELISA for EGFR binding to targeted AuNPs. (A) shows the ELISA signal (reflecting EGFR binding) normalized by nanoparticle concentration. Both C225 and VHH-122 have high binding, while PEG has almost none. Differences in binding between all three nanoparticle types are statistically significant. (B) shows the ELISA signal normalized by antibody concentration. Here, C225 has much higher signal than VHH-122, consistent with its higher binding affinity and lower number of antibodies per nanoparticle.

Binding of the nanoparticle surface-immobilized C225 and VHH-122 antibodies to soluble EGFR extracellular domain was demonstrated by ELISA in suspension. Results of the ELISA (see Fig 2) were normalized to both nanoparticle concentration and antibody concentration. As expected, C225 showed the highest level of binding, followed by VHH-122. PEG-AuNPs showed almost no EGFR binding. When normalized to nanoparticle concentration, the VHH-AuNPs demonstrated almost as much EGFR binding as C225, but when normalized to antibody concentration, C225-AuNPs showed much higher binding per antibody.

The C225-AuNPs have five times fewer antibodies attached to the surface, so each C225 antibody is much better at binding to EGFR than each VHH-122 antibody. This is consistent with the previously reported binding affinities of these two antibodies. In order to compare the binding affinity seen here on the surface of gold nanoparticles with the published binding affinity, we can calculate the expected fraction of each antibody that should be bound to EGFR using theoretical binding affinities and the known concentrations of EGFR and antibodies in solution. The KR_D_ R(equilibrium dissociation constant) can be calculated using the following formula:

$$K_{D}=\frac{\left[A][B\right]}{\left[AB\right]}$$

where [A] is the concentration of the unbound ligand (EGFR) in solution, [B] is the concentration of the unbound antibody (VHH-122 or C225) and [AB] is the concentration of bound EGFR-antibody complexes. Solving this equation with a total EGFR concentration of 10 nM, total calculated C225 concentration of 1.1 nM, and total calculated VHH-122 concentration of 3.7 nM (see S1 Dataset), we find that the saturation of C225 (ratio of bound C225 to total C225) should be ~98% under these experimental conditions, while the saturation of VHH-122 should be ~19%. This shows that the measured ELISA signal (normalized by number of antibodies) for C225 antibodies should theoretically be ~5.2x higher than the ELISA signal for the VHH-122 antibodies under these conditions. Our actual ELISA result showed that the C225 group had ~5.9x higher signal than the VHH-122 group, which is consistent with the theoretical calculation. This suggests that the antibodies on the surface of the gold nanoparticles maintain a similar binding affinity to the antibodies prior to PEGylation and surface immobilization, and that the surface immobilization or PEGylation has not affected the relative ability of the two antibody types to bind to free EGFR in solution.

### In vitro cell targeting

**Fig 3:**
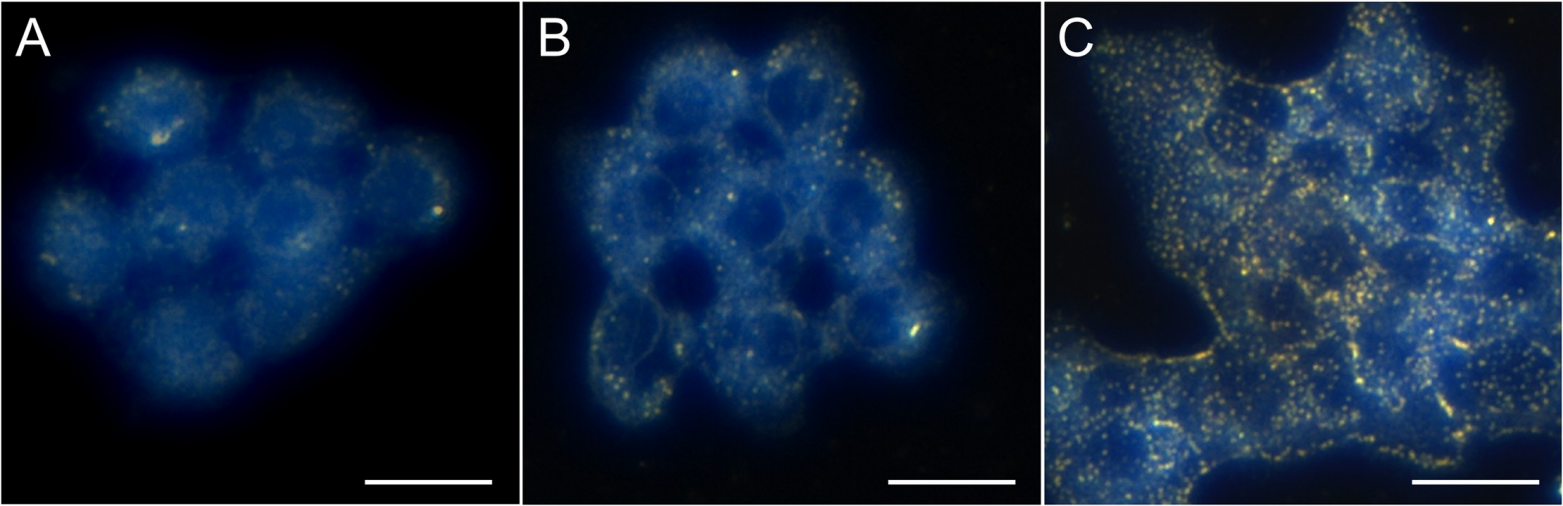
Darkfield images of AuNPs bound to A431 cells (high EGFR expression). AuNPs appear as bright yellow-orange dots in the dark field images. PEG-AuNPs (A) showed little to no cell binding, VHH-AuNPs (B) showed a moderate level of cell binding, while C225-AuNPs (C) had a high number of nanoparticles bound to the cells in culture. This demonstrates effective EGFR-targeting, particularly for the C225-AuNPs. Scale bars are 30 **μ**m.

Following incubation with gold nanoparticles, A431 cells (high EGFR expression) were imaged by darkfield microscopy to visualize gold nanoparticles binding to the cells. Darkfield images are shown in Fig 3. PEG-AuNP binding to cells was negligible. VHH-AuNPs had a moderate level of binding, but much less than the C225-AuNPs. The C225-AuNPs had a high level of binding, with bright nanoparticles visible across most of the surface of the cells under darkfield microscopy.

**Fig 4:**
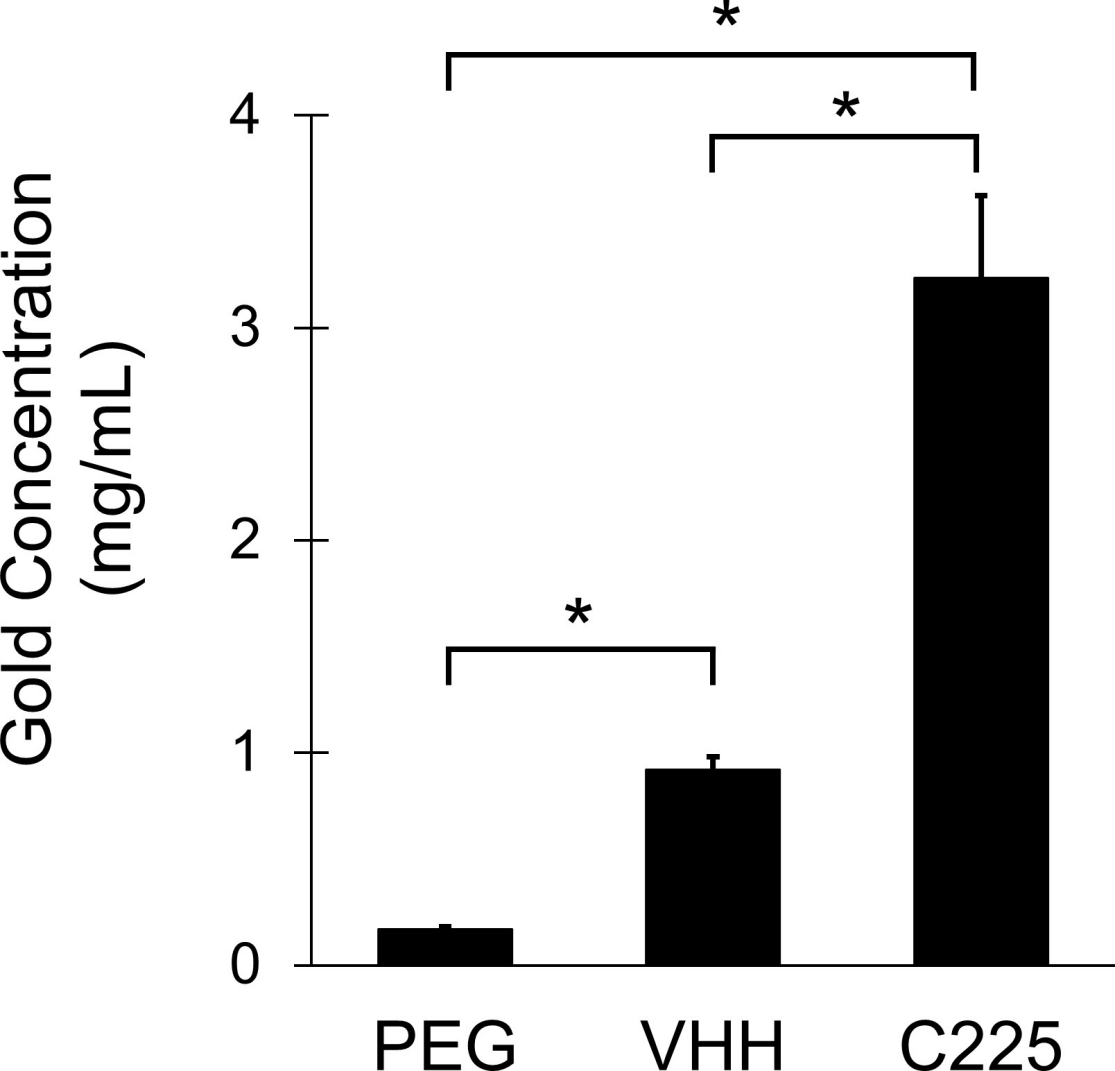
ICP-OES measurements of cell pellet gold concentrations. C225-AuNPs showed the highest degree of A431 cell binding, followed by VHH-AuNPs. PEG-AuNPs showed only minimal cell binding. All three groups were statistically different from one another.

Gold nanoparticle binding to cells was quantified by ICP-OES, which can accurately measure gold concentration in solution after acid digestion of the sample. After collecting cell pellets from each group, the gold concentration within the cell pellet was analyzed. ICP-OES results are shown in Fig 4. The cells incubated with C225-AuNPs showed very high gold concentration (3.2 mg gold/mL in the pellet), while the VHH-AuNP group showed a relatively lower gold concentration (0.9 mg/mL). However, this value was still much higher than the PEG-AuNPs, which had a gold concentration of 0.2 mg/mL. The VHH-AuNP group had 3.5x less gold bound to the cells than the C225-AuNP group, which is less than expected based on the results of the ELISA measurement of EGFR-binding per nanoparticle. Although VHH-122 has lower binding affinity to EGFR than the C225 antibody, the VHH-AuNPs should theoreticaly be capable of binding to A431 cells almost as well as the C225-AUNPs (~1.3x less in the ELISA experiment), because the VHH-AuNPs have more antibody bound to their surface. However, this result appears to contradict that hypothesis. Although VHH-AuNPs can bind soluble EGFR almost as well as C225-AuNPs, they have a reduced ability to attach (or remain attached during rinsing) to cells expressing EGFR compared to C225-AuNPs. However, compared to PEG-AuNPs, the VHH-AuNPs still demonstrate significantly higher cell binding in vitro.

### CT biodistribution study

**Fig 5:**
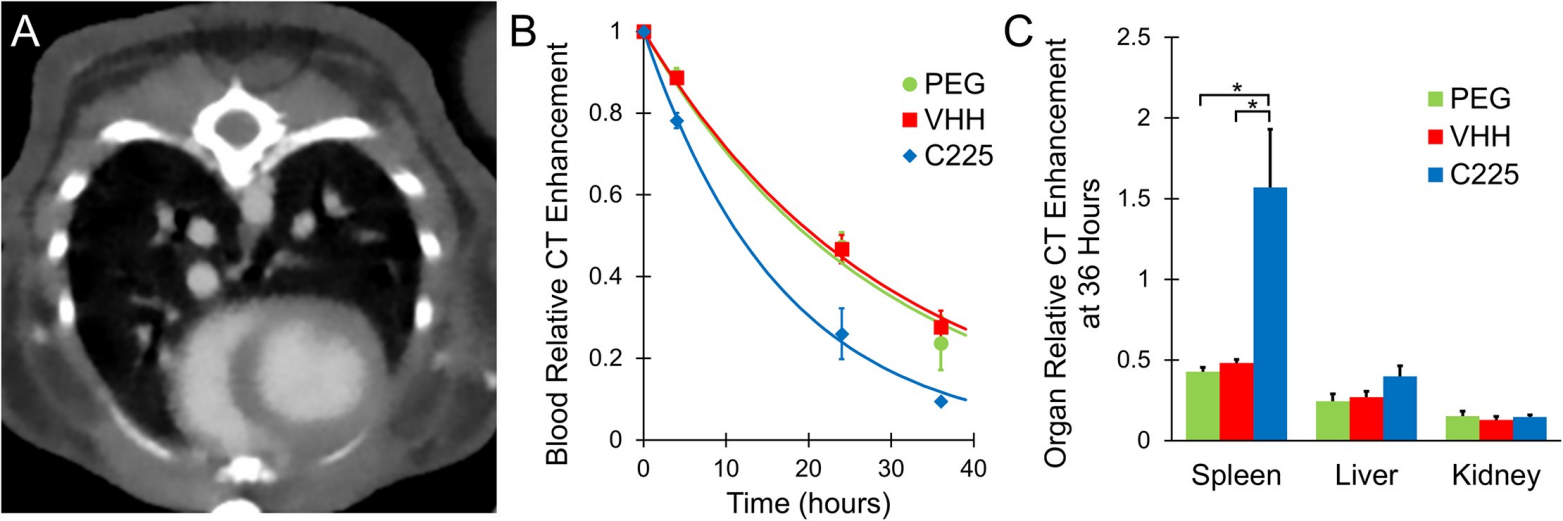
CT enhancement in healthy mice after injection of AuNP contrast agents. (A) shows a CT axial cross-section through the heart immediately after injection of VHH-AuNPs, demonstrating high CT contrast in the blood after injection. (B) shows the measured enhancement of each contrast agent in the blood over time, along with the exponential fit curve for each nanoparticle type. Enhancement at each time point is normalized to the initial enhancement immediately after injection. All nanoparticles showed an exponential decrease in concentration. C225 had a significantly shorter half-life than VHH-122 or PEG. (C) shows the organ enhancement at 36 hours (relative to the enhancement in the blood immediately after injection) for each contrast agent. C225 showed significantly more spleen accumulation than VHH-122 or PEG. All other differences were not statistically significant.

Each of the three nanoparticle types (C225-AuNPs, VHH-AuNPs, and PEG-AuNPs) was injected intravenously into healthy C57BL/6 mice (n = 3 for each group) to study the contrast agent effectiveness, biodistribution, and blood half-life. Results are shown in Fig 5. All of the nanoparticle types exhibited high CT contrast (up to 550 HU enhancement in the blood), as shown in the axial CT slice through the heart in Fig 5. Measurements of CT enhancement in the blood over time are also shown in Fig 5. After measuring the enhancement at multiple time points for each mouse, the data were fit to exponential curves to estimate the blood residence half-life. C225-AuNPs had a significantly lower calculated blood half-life (~11 hours) compared to the VHH-AuNPs and PEG-AuNPs (~20 hours for both). The VHH-AuNPs had no decrease in blood half-life compared to PEG-AuNPs, despite the presence of antibody domains on the surface of the AuNPs. Because the VHH domains lack the Fc portion, they appear to avoid rapid clearance, while the full-sized antibodies (C225) are recognized and cleared from the bloodstream much more quickly, as we originally hypothesized. The measured enhancement in the spleen 36 hours after contrast injection was significantly higher for the C225-AuNPs than for the other groups, while the concentrations in the liver and kidney were similar between groups. This high spleen enhancement at 36 hours further supports the idea that C225-AuNPs are recognized by phagocytes within the reticuloendothelial system and cleared from the blood (and into the spleen) more rapidly than the other groups.

### CT tumor imaging

**Fig 6:**
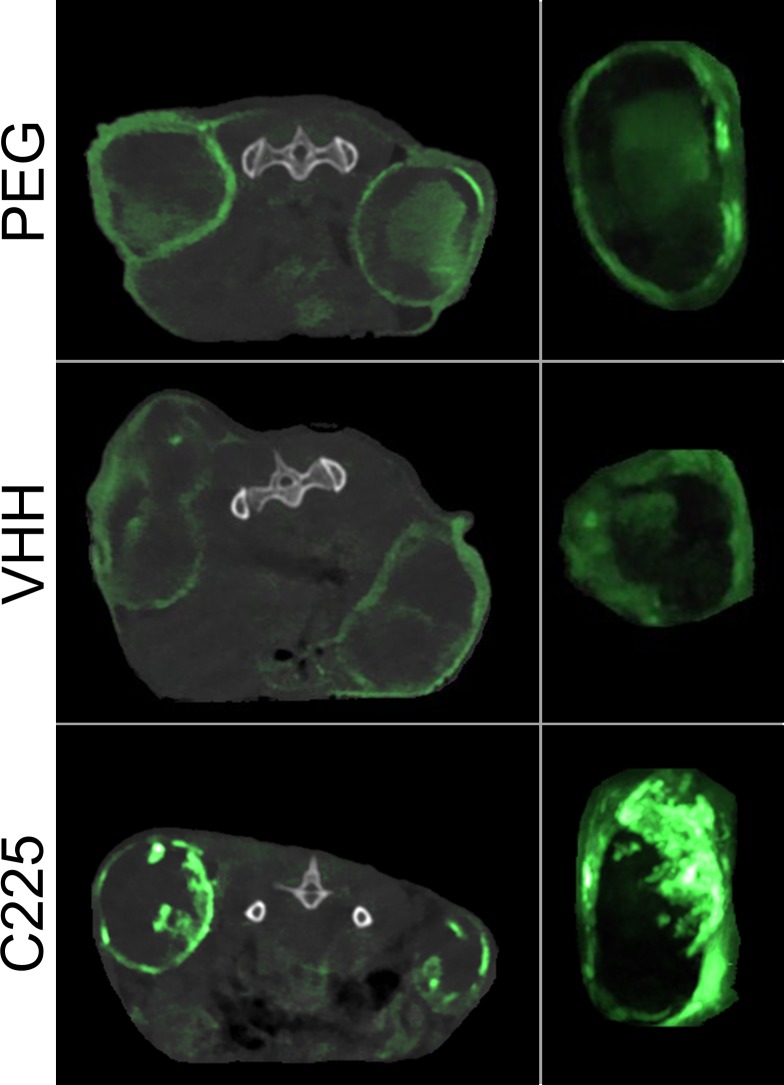
Dual-energy CT slices of tumors 48 hours post-injection. Axial CT slices of the mice through the center of the tumors are shown on the left, while a coronal maximum intensity projection (MIP) of an isolated tumor from each mouse is shown on the right. Each MIP includes 20 slices through the center of the tumor. Each image is an overlay of the single energy CT scan (in grayscale) and the dual-energy gold concentration map (in green). Gold nanoparticles were cleared from the bloodstream at this time point and accumulated predominately within the well-perfused tumor margins. The relatively poorly perfused tumor cores showed low nanoparticle uptake. All tumors had substantial nanoparticle accumulation, but C225-AuNPs showed the highest tumor enhancement. The single energy CT scans are windowed from -500 to 2000 HU for the axial slices and 0 to 3000 HU for the coronal MIPs while the dual-energy decomposition gold maps are windowed from 1.5 to 20 mg/mL for the axial slices and 0.5 to 20 for the coronal MIPs.

Targeted (C225 and VHH-122) and control (PEG) nanoparticles were injected into nude mice (n = 8 for each group) with bilateral subcutaneous A431 cell line tumors, which demonstrate high levels of EGFR expression. Dual-energy CT was used to visualize and quantify gold within each of the tumors. The mice were imaged 48 hours after contrast agent injection, at which point most of the contrast had been cleared from the bloodstream. Fig 6 shows CT images (with gold concentration shown in green) of the flank tumors in mice for each of the three groups including both an axial thin section of the whole mouse and a coronal thick section of an isolated tumor. All of the tumors (even those in the control PEG-AuNP group) had gold accumulation due to the EPR effect. The VHH-AuNPs and PEG-AuNPs had no noticeable difference in accumulation in the CT images, but the C225-AuNPs appear to have much higher gold accumulation within the tumors.

**Fig 7:**
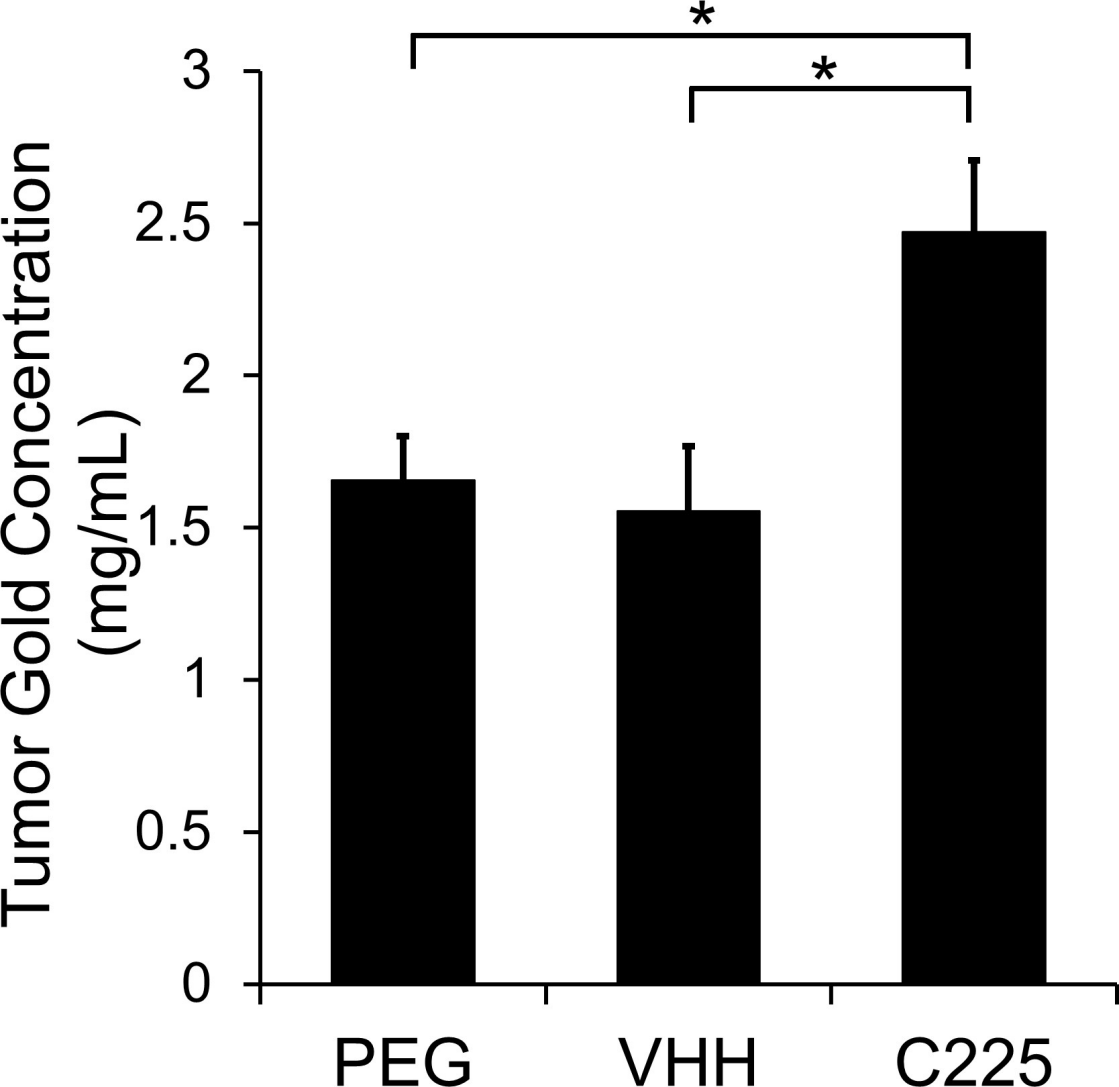
CT quantification of gold nanoparticle accumulation in the tumor margins. Enhancing regions of each tumor were segmented and the average gold concentration within each segmented tumor was calculated. C225-AuNPs have significantly higher accumulation in the enhancing portions of the tumor than either VHH-AuNPs or PEG-AuNPs. Targeting with VHH-122 did not provide any increase in accumulation relative to the PEG controls.

Fig 7 shows the quantification of average gold concentration within the enhancing regions of the tumors for each group. Consistent with the CT images, the measured gold accumulations in the VHH-AuNP and PEG-AuNP groups are statistically indistinguishable from one another. The C225-AuNP group, on the other hand, has significantly higher gold accumulation than either of the other two groups. These results contradicted our original hypothesis that the shorter blood residence time for C225-AuNPs would lead to less total tumor uptake of the nanoparticles, and therefore lower gold concentrations. We also saw that the relatively lower EGFR binding affinity of the VHH-AuNPs was not effective enough in vivo to increase the gold accumulation beyond the accumulation from EPR alone. The strong affinity of C225-AuNPs for binding to A431, on the other hand, led to significant additional accumulation.

## Discussion

In this study we examined the use of three different contrast agents for imaging EGFR-expressing tumors by CT. All of the imaging agents showed effective CT contrast, with high initial enhancement within the blood and high contrast within the tumors and other organs (spleen, liver, kidney) at later time points. The first contrast agent, PEG-AuNPs, demonstrated a long blood half-life (~20 hours) due to a robust PEG coating which minimized surface protein adsorption and subsequent immune recognition and clearance. The PEG-AuNPs contained no targeting ligand and could only accumulate in tumors due to the leaky vasculature within the tumors (EPR effect). All the tumors in the PEG-AuNP group had a moderate level of gold nanoparticle uptake, showing the inherent effectiveness of EPR in this tumor model.

The second contrast agent, VHH-AuNPs, had many (~25) small VHH-122 antibodies on the surface of each nanoparticle. These domains have moderately high binding affinity for EGFR, as demonstrated by the effective binding of soluble EGFR to VHH-AuNPs in solution. The domains are small and lack the Fc portion of the antibody, which limits the ability of phagocytic cells to recognize these nanoparticles and engulf them. The VHH-AuNPs had a long blood half-life (~20 hours), which was indistinguishable from the PEG-AuNP blood half-life, showing that the presence of single-domain antibodies did not interfere with the stealth nature of these nanoparticles. However, these nanoparticles demonstrated less binding affinity for A431 cells in vitro compared to the full-sized C225 antibody. The binding of VHH-AuNPs to A431 cells was successfully demonstrated in vitro, but it was not sufficient to increase the accumulation of these targeted nanoparticles in vivo.

The third contrast agent, C225-AuNPs, contained several (~5) full-sized humanized anti-EGFR antibodies conjugated to the nanoparticle surface. These clinically-used antibodies have very high binding affinity for EGFR, as demonstrated by effective in vitro binding both to soluble EGFR in solution and to EGFR-expressing A431 cells in culture. These targeted nanoparticles demonstrated a reduced blood half-life (~11 hours) compared to the other two contrast agents, consistent with increased immune clearance due to the presence of full-sized IgG antibodies on the nanoparticle surface. Despite the reduced blood residence time, the C225-AuNPs had significantly higher tumor uptake than the other two contrast agents. The high EGFR binding affinity of the C225-AuNPs was more than enough the counteract the effects of the reduced blood residence time on total tumor accumulation.

Our original hypothesis that VHH-122 antibodies would provide better targeting than full-sized antibodies was not supported by the data. Even though the C225-AuNPs had lower blood half-life (and therefore, less time to accumulate in the tumors), they demonstrated significantly higher tumor accumulation than any of the other nanoparticle types. Surprisingly, the VHH-AuNPs showed no higher accumulation in tumors than the PEG-AuNPs. The VHH-AuNPs showed effective EGFR binding when the EGFR was in solution but showed decreased binding when the EGFR was on the surface of A431 cells. In previous studies with VHH domains[25], the VHH-122 antibody was equally able to bind to EGFR in solution (from cell lysates) and to EGFR-expressing cell lines (including A431 cells). However, we saw a decrease in binding efficacy when binding to cells compared to binding to free EGFR in solution. We hypothesize that this reduced binding efficacy is due to the presence of the relatively large gold nanoparticles attached to the antibodies. When free VHH-122 is bound to EGFR-expressing cells in culture, the binding strength is high enough to prevent the antibody from being released during rinsing steps. However, when the antibody-targeted AuNPs are attached to cells in culture, the antibody binding strength must be high enough to keep the relatively massive gold nanoparticle attached and prevent the nanoparticle from being sheared away from the cells during rinsing steps. This requires a much higher overall binding strength because of the large size (and mass) of the gold nanoparticles relative to the size of the individual antibodies. This result was further supported by the in vivo study, which showed no increase in nanoparticle accumulation as a result of VHH-122 targeting (compared to non-targeted controls). Our hypothesis was that the increased avidity of the VHH-AuNPs due to the presence of many antibodies (~25 per nanoparticle) on surface could potentially overcome this issue, but that was not the case. It is likely that only a small fraction of the VHH-122 antibodies on the surface of the nanoparticle could actually interact with the EGFR on the A431 cells due to the high curvature of the AuNP surface.

Although one of the primary determinants of tumor accumulation for nanoparticles is the nanoparticle blood residence time, this is clearly not the only important factor in determining total tumor accumulation. In this case, we saw that an antibody-targeted nanoparticle contrast agent with high binding affinity for the target cells can have much higher total tumor accumulation than a non-targeted nanoparticle even if it has a significantly lower blood residence time. The C225 antibody, with high affinity and reduced blood residence time was superior to the VHH-122 antibody, a tumor targeting ligand with lower affinity but a long blood residence time. When making the choice for a tumor targeting ligand, it is important to consider both the effect that the targeting ligand will have on the nanoparticle clearance and the overall targeting efficacy of the targeting ligand. For attachment to nanoparticles, these data suggest that high affinity ligands are preferable for in vivo use, even if they moderately reduce blood residence time. Although these data suggest that ligand affinity has more impact on tumor accumulation than blood half life under these conditions, the effects of these two parameters could be confirmed by expanding this study with additional controls. The ideal controls in this case would be nanoparticles conjugated with non-specific IgG antibodies and nanoparticles with non-specific VHH antibodies (not targeted to EGFR). These control nanoparticles would have similar pharmacokinetics to the corresponding targeted nanoparticles, which would allow for analysis of the effects of affinity and pharmacokinetics isolated from one another.

The C225-AuNPs used in this study showed highly effective tumor targeting relative to non-targeted controls. This demonstrates the feasibility of using targeted nanoparticle agents for the detection of surface receptors upregulated on tumor cells, including tumors overexpressing EGFR. The ability to detect tumor EGFR expression (based on the degree of tumor CT enhancement) could be extremely useful in discriminating between benign and malignant lung tumors on CT exam. This can be used to better predict which patients with an identified lung nodule are in need of biopsy and potential surgery and which patients can instead be treated with watchful waiting and periodic follow-up scans. Such methods to improve non-invasive discrimination between benign and malignant nodules could have a large impact on patient well-being by reducing unnecessary invasive procedures.

A previous small-scale study[19] initially demonstrated the use of C225-targeted AuNPs for CT imaging. That study showed that C225-targeted AuNPs have higher tumor enhancement than non-targeted controls, as we confirmed in this study. However, the mice in that study were injected with a much lower dose of gold nanoparticles (5 mg gold) than in the present study (20 mg gold). The overall enhancement in the tumors was significantly less for both groups in that study, but the ratio of tumor enhancement for targeted nanoparticles to non-targeted nanoparticles was higher, due to very low non-targeted nanoparticle uptake by the tumors. In contrast, we saw moderate uptake of non-targeted AuNPs in this study. Presumably, the reduced dose of AuNPs decreased the effectiveness of non-targeted tumor accumulation (EPR only) more than it reduced the effectiveness of targeted tumor accumulation (EPR + targeting). Therefore, the specificity of the targeted nanoparticle accumulation may increase as the overall dose of nanoparticles decreases, but the sensitivity of CT for detecting tumors will also decrease with lower contrast dose. Both of these studies also used similarly-sized gold nanoparticles (30 nm and 25 nm), but it has been shown previously that smaller particles tend to demonstrate more specific targeted uptake than larger nanoparticles do[21]. The results from this study may also not extend to other in vivo applications of gold nanoparticles (SERS imaging, photoacoustic imaging) which require much smaller doses of gold nanoparticles. Optimization of both the injected dose and nanoparticle size for targeted and non-targeted nanoparticles must be performed for a given application to maximize the specificity of the targeted agent while still maintaining sufficient contrast to positively identify the target tumors.

## Conclusions

We have compared the effectiveness of three gold nanoparticle contrast agents for the imaging of EGFR-expressing tumors: C225-AuNPs, VHH-AuNPs, and non-targeted control PEG-AuNPs. The C225 and VHH-AuNPs both bind to EGFR in vitro, but the C225-AuNPs show higher binding affinity in cell culture. After in vivo injection, the VHH-AuNPs and PEG-AuNPs show equivalent tumor accumulation, while the C225-AuNPs show much higher accumulation, despite having the lowest half-life of the three groups. All three of the contrast agents could be used effectively for both vascular imaging and tumor imaging by CT, however, the C225-AuNPs showed significantly increased tumor uptake due to specific tumor targeting. This increased enhancement with targeted nanoparticles can be used to better identify receptor status of tumors and improve tumor discrimination and characterization.

## Supporting information

S1 Dataset SDS-PAGE and ELISA quantification. (XLSX)

S2 Dataset ICP-OES measurements of cell pellet gold concentration. (XLSX)

S3 Dataset CT nanoparticle biodistribution raw measurements. (XLSX)

S4 Dataset In vivo tumor gold concentration measurements. (XLSX)
